# Preclinical development of a novel CD47 nanobody with less toxicity and enhanced anti-cancer therapeutic potential

**DOI:** 10.1186/s12951-020-0571-2

**Published:** 2020-01-13

**Authors:** Linlin Ma, Min Zhu, Junwei Gai, Guanghui Li, Qing Chang, Peng Qiao, Longlong Cao, Wanqing Chen, Siyuan Zhang, Yakun Wan

**Affiliations:** 1 Jiading District Central Hospital Affiliated Shanghai University of Medicine and Health Sciences, Shanghai, China; 2 Shanghai Novamab Biopharmaceuticals Co., Ltd, Shanghai, China; 3 XPCC Tenth Division Beitun Hospital, Beitun, Xinjiang China

**Keywords:** CD47, Immunotherapy, Nanobody, Bispecific antibody

## Abstract

**Background:**

CD47, the integrin-related protein, plays an important role in immune resistance and escape of tumor cells. Antibodies blocking the CD47/SIRPα signal pathway can effectively stimulate macrophage-mediated phagocytosis of tumor cells, which becomes a promising approach for tumor immunotherapy. Nanobodies (Nbs) derived from camelid animals are emerging as a new force in antibody therapy.

**Results:**

HuNb1-IgG4, an innovative anti-CD47 nanobody, was developed with high affinity and specificity. It effectively enhanced macrophage-mediated phagocytosis of tumor cells in vitro and showed potent anti-ovarian and anti-lymphoma activity in vivo. Importantly, HuNb1-IgG4 did not induce the agglutination of human red blood cells (RBCs) in vitro and exhibited high safety for hematopoietic system in cynomolgus monkey. In addition, HuNb1-IgG4 could be produced on a large scale in CHO-S cells with high activity and good stability. Also, we established anti-CD47/CD20 bispecific antibody (BsAb) consisted of HuNb1 and Rituximab, showing more preference binding to tumor cells and more potent anti-lymphoma activity compared to HuNb1-IgG4.

**Conclusions:**

Both of HuNb1-IgG4 and anti-CD47/CD20 BsAb are potent antagonists of CD47/SIRPα pathway and promising candidates for clinical trials.
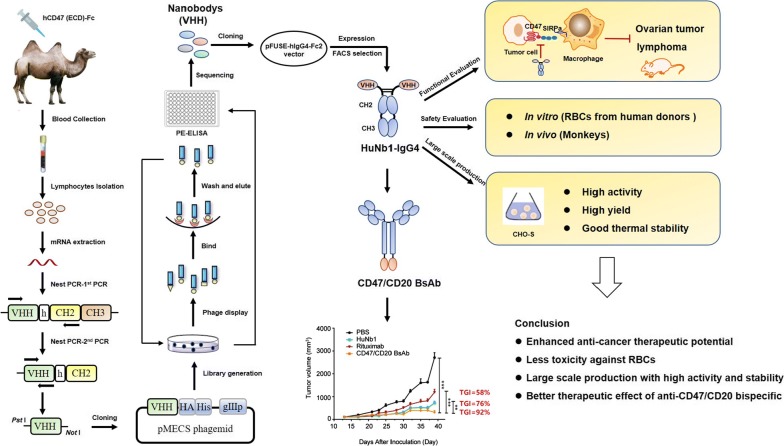

## Background

Cancer immunotherapy targeting T cell checkpoint pathways have shown striking clinical success in a variety of blood and solid tumors. However, the complete elimination of cancer cells depends not only on T-cell-mediated adaptive immunity, but also on innate immune cells such as macrophages that act as effectors killing tumor cells through cytotoxin release or by physical engulfment [1]. Not surprisingly, tumor cells have evolved mechanisms to evade the killing role by these innate immune cells, which results in resistance to immunotherapy targeting T cell checkpoint PD1/PD-L1. One of stratagems for cancer cell evasion is to increase the expression of CD47, a ubiquitously expressed cell surface receptor [2].

CD47, also known as integrin-associated protein, can interact with signal regulatory protein alpha (SIRPα) expressed primarily on phagocytic cells including macrophages and dendritic cells [3–5]. CD47-SIRPα interaction signals to inhibit phagocytosis through deactivation of myosin-II-associated machinery required for the engulfment [6]. Additionally, blockade of CD47-SIRPα interaction has been shown to enhance antitumor T-cell immune responses [7–9].

Increased expression of CD47 has been observed in multiple blood and solid tumors including acute myeloid leukemia (AML), non-Hodgkin lymphoma (NHL), gastric cancer, ovarian cancer, colon cancer, hepatocellular cancer and other tumor cells. Furthermore, CD47 overexpression has been shown to correlate with poor clinical outcome [10–12]. Blockade of CD47-SIRPα interaction increases macrophage-mediated phagocytosis and enhances tumor elimination in a variety of preclinical models. In addition, two CD47-targeting monoclonal antibodies (mAbs) and a receptor fusion protein (SIRPα-Fc) are currently undergoing clinical trials, which showed positive results. However, these antibodies have been reported to cause platelet aggregation and red blood cells (RBCs) hemagglutination [13–15]. These adverse effects are associated with high expression of CD47 on RBCs, especially aged RBCs and blockade of CD47 on RBCs can lead to macrophages-mediated phagocytic removal [16]. Thus, the novel therapy targeting CD47 with less adverse effects is still needed to be developed.

Nanobodies (Nbs) are a novel type of single-domain antibody fragments derived from naturally-occurring heavy-chain IgG antibodies [17]. Due to their small size (~ 12 kDa), high affinity and high stability, Nbs have been recognized as ideal building blocks for the development of novel biological drugs compared to conventional mAbs. Nbs are also easy to be modified for the different use, which makes Nbs ideal therapeutic reagents [18–20]. To reduce adverse effects of blocking CD47-SIRPα interaction, we herein described generation and characterization of HuNb1-IgG4, an anti-CD47 Nb fusion protein with low affinity for RBCs. Our results show that HuNb1-IgG4 enhances macrophage-mediated phagocytosis and shows potent anti-tumor activities in vivo. More importantly, HuNb1-IgG4 treatment does not cause platelet aggregation in human RBCs and shows high safety for RBCs in the monkey. In order to further improve HuNb1-IgG4 safety and efficacy, we also established anti-CD47/CD20 bispecific antibody (BsAb) consisted of HuNb1 and Rituximab, showing more preference binding to tumor cells and more potent anti-lymphoma activity compared to HuNb1-IgG4.

## Materials and methods

### Cell lines

HEK-293F, CHO-S, Raji, SK-OV-3 and Jurkat E6.1 cells were obtained from the American Type Culture Collection (ATCC). HEK-293F cells and CHO-S cells were cultured in FreeStyle™ 293 or FreeStyle™ CHO expression medium (Invitrogen, Carlsbad, CA, USA) respectively, supplemented with 1% Penicillin–Streptomycin (10,000 U/mL) (Invitrogen). Raji cells and SK-OV-3 cells highly expressing CD47 were grown in RPMI1640 containing 10% FBS (Gibco, GrandIsland, NY, USA) and 1% Penicillin–Streptomycin. Jurkat E6.1 cells were cultured in RPMI1640 supplemented with 10% heat-inactivated fetal calf serum (FCS) (Biological Industries, Kibbutz Beit Haemek, Israel), 1% Penicillin–Streptomycin and 2 mM L-glutamine (Gibco).

### Expression and purification of hCD47 (ECD)-Fc and hSIRPα (ECD)-Fc

hCD47 ectodomain (ECD) (20–136) or hSIRPα (ECD) (31–373) fused to human Fc fragment were amplified by PCR and cloned into pFUSE vector (Invitrogen). hCD47 (ECD)-Fc and hSIRPα (ECD)-Fc were expressed using PEI-mediated transient transfection system in HEK-293F cells. The supernatants were collected after 5 days and hCD47 (ECD)-Fc and hSIRPα (ECD)-Fc proteins were purified with protein A affinity chromatography.

### Immunization and library construction

A young healthy male Bactrian camel was firstly immunized with hCD47(ECD)-Fc mixed with an equal volume of Freund’s complete adjuvant (Sigma-Aldrich, StLouis, MO, USA), and then continued to accept immunization with Freund’s incomplete adjuvant (Sigma-Aldrich) once a week. After the seventh immunization, peripheral blood lymphocytes (PBLs) were separated from 100 mL camel blood by Ficoll-Paque PLUS (GE Healthcare, Pittsburgh, PA, USA) and a phage display library containing the genes coding for the variable domains of the heavy-chain antibodies (VHH) was then generated according to established methods [21, 22]. All procedures were conducted according to the National Institutes of Health guide for the care and use of Laboratory animals.

### Nbs selection, expression and purification

Nbs specific binding to human CD47 were screened through phage display and Periplasmic Extract ELISA (PE-ELISA), according to previously described procedure [21, 23]. After sequencing the selected clones, the VHH fragments were amplified and subcloned into pFUSE-hIgG4-Fc2 vector (Invivogen, San Diego, CA, USA). The recombinant vector was transfected into CHO-S cells, and Nb-Fc proteins were purified by protein A affinity chromatography. For prokaryotic expression and purification, VHH fragments cloned in pMECS were transformed into non-suppressor strain WK6 cells, and purified using affinity chromatography on a Ni-sepharose resin column (GE Healthcare). The purity of Nbs was detected by size exclusion chromatography-high performance liquid chromatography (SEC-HPLC).

### Preparation of B6H12, Hu5F9-G4, Rituximab and CD47/CD20 BsAb

For preparation of positive control antibodies including B6H12, Hu5F9-G4 or Rituximab (CD20 mAb), the DNA sequences of their heavy chains and light chains were synthesized and cloned into pcDNA3.1+ vector respectively. The recombinant vectors were transfected into HEK-293F cells simultaneously, and the antibodies were purified by protein A affinity chromatography. For preparation of CD47/CD20 BsAb, the DNA sequence of anti-CD47 Nb was linked to the DNA sequence of heavy chain of Rituximab. The recombined heavy chain and light chain of Rituximab were amplified and cloned into pcDNA3.1+ vector, and its expression and purification were similar to that of B6H12, Hu5F9-G4 and Rituximab.

### Activity assay of Nb-IgG4 binding to CD47 and blocking CD47-hSIRPα

To determine the activity of anti-CD47 Nb-IgG4 binding to CD47 on the surface of cells, Raji cells highly expressing CD47 were harvested and incubated with a series concentration of Nb-IgG4 antibodies, with anti-CD47 mAb B6H12 as the control. FITC conjugated anti-human Fc (eBioscience, San Diego, CA, USA) was used as a secondary antibody. To determine the activity of Nb-IgG4 blocking CD47-hSIRPα, Raji cells were incubated with the purified hSIRPα (ECD)-Fc labeled with biotin and a gradient concentration of anti-CD47 antibodies, followed by staining with streptavidin-PE (eBioscience). The signals were detected by BD FACS Calibur instrument (BD Biosciences, Franklin Lakes, New Jersey, USA) and data were analyzed using Flowjo software.

### Affinity determination of Nb-IgG4 to hCD47

The kinetics of Nb-IgG4 binding to recombinant hCD47 antigen was determined by Biofilm interferometry (BLI) measurement with a Fortebio Octet Red 96 instrument (ForteBio, Inc., Menlo Park, CA, USA). Briefly, the diluted Nb-IgG4 or B6H12 (5 μg/mL) were coupled to protein A sensors and then incubated with a series diluted hCD47 (ECD)-Fc, followed by dissociation in PBST buffer. The binding curves were fit in 1:1 global fitting mode by Octet analyze software. The association and dissociation rate were monitored and the equilibrium dissociation constant (KD) was calculated.

### Specificity analysis of Nb binding to CD47 expressed on the different species

The mouse CD47 (ECD)-Fc and rat CD47 (ECD)-Fc were expressed and purified in HEK-293F cells. 5 μg/mL proteins (human CD47 (ECD)-Fc, mouse CD47 (ECD)-Fc, rat CD47 (ECD)-Fc and Fc) were coated onto microtiter plates overnight at 4 °C. After blocked with 1% bovine serum albumin (BSA) at room temperature for 2 h, 10 μg/mL Nb was added and incubated at room temperature for 1 h. Next, the plates were incubated with mouse anti-HA antibody (Covance, Princeton, NJ, USA) for 1 h, followed by incubating with anti-mouse IgG-alkaline phosphatase (Sigma-Aldrich) for another 1 h. The absorbance at 405 nm was read on Enspire (PerkinElmer, Waltham, MA, USA) after addition of the chromogenic solution containing bisphosphate (pNPP) (Sigma-Aldrich).

### RBCs binding activity and aggregation assay

Fresh human blood samples were provided by six healthy donors, and RBCs were separated from the whole bloods through centrifugation. After washed twice with PBS, RBCs were diluted to generate cell suspension. For RBCs binding activity assay, RBCs were incubated with a series concentration of Nb-IgG4 antibodies, with Hu5F9-G4 as the control. FITC conjugated anti-human Fc were used as a secondary antibody and the signals were quantified by FACS. For RBCs aggregation assay, 50 μL 2% (v/v) RBCs suspension were mixed with an equal volume of antibodies serially diluted by fourfold in normal saline in a U bottom 96-well plate, with 50 μL normal saline as RBCs control. RBC aggregation was observed after incubation at room temperature for 40 min.

### Macrophage-mediated phagocytosis assay

Normal human peripheral blood mononuclear cells (PBMCs) were isolated using Ficoll-Paque Plus (GE Healthcare) and cultured in RPMI 1640 medium supplemented with 10% heat-inactivated FCS, 1% Penicillin–Streptomycin and 40 ng/mL human macrophage colony-stimulating factor (M-CSF) (Novaprotein, Summit, NJ, USA) for 9–11 days. Thereafter, non-adherent cells were removed by washing with PBS, and adherent cells representing macrophages were subsequently separated by TrypLE (Gibco)*.*

Macrophages were co-incubated with Jurkat E6.1 cells or Raji cells stained with 1 μM CFSE (eBioscience) in flat bottom 96-well plates with the ratio of 1:4. The tested antibodies were added and incubated with cells at 37 °C for 4 h. The cells were then rinsed five times by PBS and the macrophages were harvested by TrypLE. CFSE positive events identified as phagocytosis were quantified by FACS.

### Specificity analysis of CD47/CD20 BsAb binding to CD47 and CD20

To determine the specificity of CD47/CD20 BsAb binding to CD47 or CD20 expressed on Raji cells, Raji cells was co-incubated with HuNb1-biotin or Rituximab-biotin as well as antibodies including Rituximab (CD20 mAb), HuNb1 (CD47 Nb) or CD47/CD20 BsAb, with IgG4 as negative Ab. Cells were stained with streptavidin-PE, and the signals were detected by FACS.

To determine whether CD47/CD20 BsAb selectively bind to Raji cells in the presence of tenfold excess RBC. 3E6 RBC and 3E5 Raji cells were incubated with CD47/CD20 BsAb at flat bottom 96-well plate, with only 3E5 Raji cells incubated with CD47/CD20 BsAb as the control. The goat anti-human IgG-FITC was added and incubated with cells at 37 °C for 4 h. FITC positive events on Raji cells and RBCs were quantified by FACS.

### Xenograft models

To establish the human lymphoma model, six-eight weeks old female NOD/shi-scid/γc^null^ (NOG) mice provided by Vital River Laboratory Animal Center (Beijing, China) were injected by tail vein (i.v.) with Raji cells at a concentration of 5E6 cells in 0.2 mL PBS. Treatment was started 12 days after tumor engraftment, with a daily i.p. given of Nb-IgG4 (20 mg/kg) or PBS for four weeks. For the human ovarian cancer model, six-eight weeks old female BALB/c nude mice (Vital River Laboratory) were subcutaneously injected at the right flank with 5 × 10^6^ SK-OV-3 cells resuspended in 0.2 mL PBS mixed with Matrigel Matrix (Corning) with a ratio of 1:1. When tumor volumes reached 50–100 mm^3^, the mice were randomly into five groups: PBS as a negative control, Hu5F9-G4 as a positive control, Nb-IgG4 0.2 mg/kg, 1 mg/kg and 5 mg/kg as three experimental groups. Antibodies were given i.p. daily for a total of 12 days.

Perpendicular dimensions were measured using a vernier scale caliper twice per week, and the tumor volumes were calculated according to the following equation:

$${\text{Tumor volume }}\left( {{\text{mm}}^{3} } \right) = 1/2 \times \left( {{\text{length}}} \right) \times \left( {{\text{width}}} \right)^{2} .$$

All procedures were conducted according to the National Institutes of Health guide for the care and use of Laboratory animals. The mice experiments were approved by the Animal Experimental Ethics Committee of Shanghai University of Medicine and Health Sciences.

### Hematoxylin and eosin (H&E) staining

H&E staining was performed on thin sections of ovarian tumor obtained from mice treated with PBS, Hu5F9-G4 or Nb-IgG4. The tumor tissue was fixed in 10% neutral buffered formalin (NBF) and embedded in paraffin. Paraffin sections were stained by H&E for histopathological analysis. The images were obtained using an inverted light microscope (Leica, Wetzlar, Germany).

### Immunohistochemistry (IHC)

Chromogenic IHC was performed on thin sections of tumor obtained from mice treated with PBS and Nb-IgG4. The sections were stained with a rabbit polyclonal antibody against F4/80 (Abcam). F4/80 detection was performed using horseradish peroxidase (HRP)-conjugated goat anti-rabbit IgG secondary antibody, followed by colorimetric detection using diaminobenzidine (DAB; Promega, Mullion, WI, USA). The images were obtained using an inverted light microscope (Leica, Wetzlar, Germany).

### Toxicity study in non-human primates

Female monkeys were raised in single stainless-steel cages with suitable specifications. Except for the special cases that required fasting, the animals were fed twice a day in the morning and afternoon, with an average of about 100 grams of feed per animal. During the period of quarantine/domestication and testing, animals drunk water freely. Each animal's chaplet was labeled with a metal tag indicating the ID number, group, lab animal number and sex. Monkeys were housed in a temperature-controlled (22 ± 4 °C) and humidity-controlled environment (40% to 70%) on a 12 h light/dark cycle with free access to food and water.

For single-dose experiment, one monkey enrolled in the low-dose group was given 10 mg/kg Nb-IgG4, and another monkey enrolled in the high-dose group was given 30 mg/kg Nb-IgG4. For two-dose experiment, two monkeys were given 3 mg/kg Nb-IgG4 at Day 1, and injected with 60 mg/kg or 200 mg/kg Nb-IgG4 at day 8. Intravenous infusion with an injection pump was administered at a given rate of 0.5 mL/kg/min for approximately 10 min. The blood samples were collected at 1 days, 2 days, 3 days, 5 days, 8 days, 12 days, 15 days and 22 days respectively. The parameters from blood samples including hemoglobin (HGB) levels and RBC counts were recorded. The monkey experiments were approved by the Animal Experimental Ethics Committee of Shanghai University of Medicine and Health Sciences.

### Thermostability analysis

SEC-HPLC was employed to analyze thermostability of Nbs. Briefly, the selected Anti-CD47 Nb was diluted to 1 mg/mL and incubated at 25 °C and 40 °C respectively for 0, 2, 4, 6, 8, 10 days. After incubation, the stability of Nb was measured by SEC-HPLC analyses.

### Statistical analysis

Statistical analyses were performed using Graphpad Prism version 6 software. Data are expressed as the mean ± SEM or mean ± SD. The statistically significant differences between groups were performed by one-way ANOVA or two-way ANOVA with Holm-Sidak multiple comparisons test. *P* < 0.05 was considered to be significant.

## Results

### Anti-human CD47 specific Nbs screened from a phage display library

**Fig. 1:**
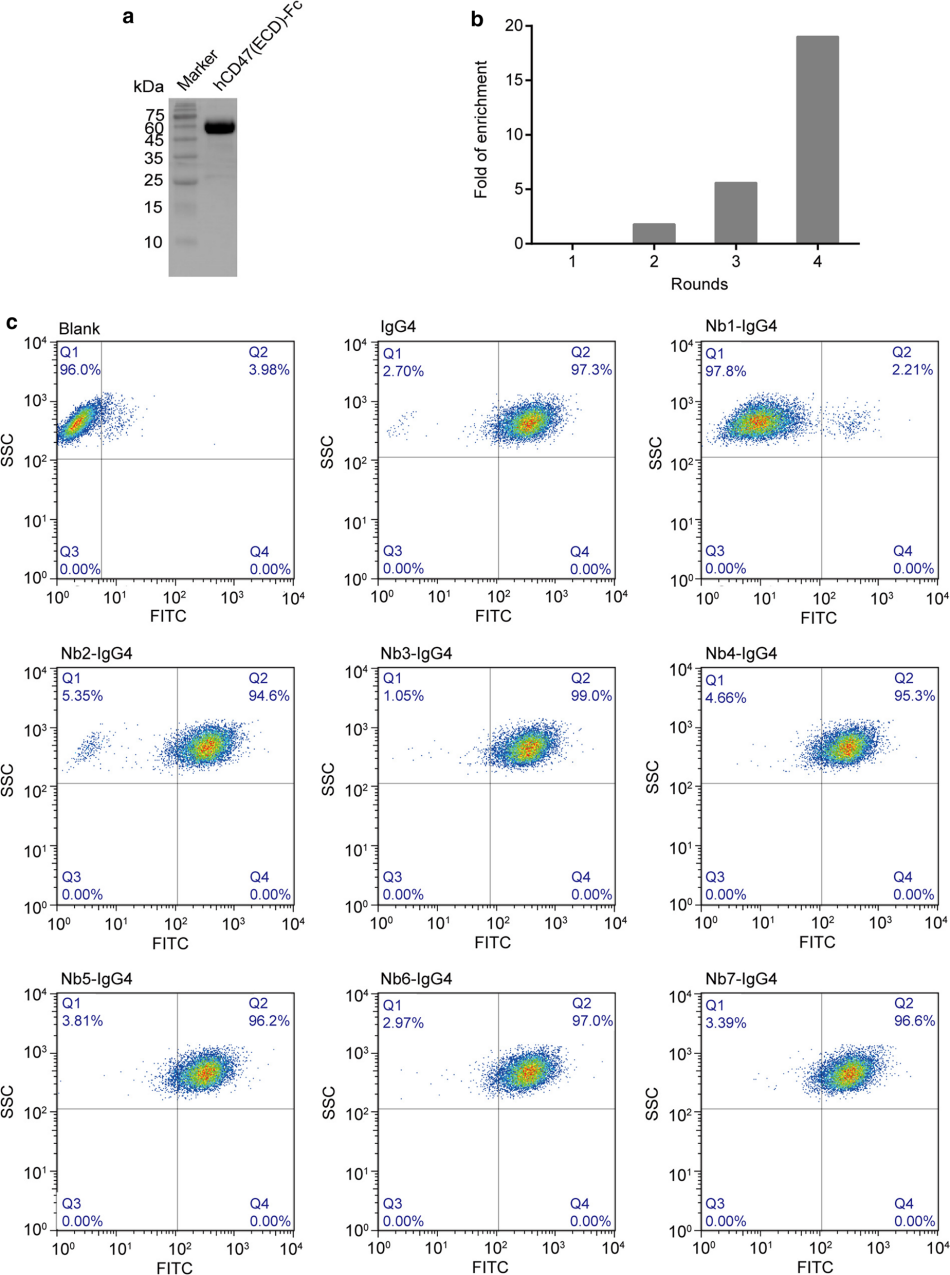
Anti-CD47 nanobody discovery. **a** SDS-PAGE of purified CD-47 ectodomain (ECD) antigen. Lane 1 was loaded with protein ladder; Lane 2 showed purified human CD47 (ECD)-Fc antigen. The samples were separated by using 10% gradient PAGE gels and stained with Coomassie blue. The experiment was performed in triplicate and one representative experiment was shown. **b** The enrichment fold for phage particles in wells coated with antigen versus wells without antigen was detected after each round of panning. The experiment was performed once. **c** The blocked nanobody was selected by FACS. The Nb1-IgG4 was identified that could block the interaction between human CD47 and SIRPα. The experiment was performed in triplicate and one representative experiment was shown

In order to construct anti-CD47 Nb phage display library, CD47 ectodomain fused to Fc (CD47-ECD-Fc) was purified as antigen with high purity through HEK-293F eukaryotic expression system (Fig. 1a). After immunization and RNA extraction from PBLs, the VHHs gene were amplified through a nest PCR amplification for generating 700 bp fragments of VHH-h-CH2 and 400 bp fragments of VHH (Additional file 1: Figure S1a, b). The library insert ratio and capacity were then detected after electro-transforming the pMECS-VHH into TG1 cells. As indicated in Additional file 1: Figure S1c, the insert ratio was approximately 100% through PCR analysis, and the library capacity reached a total size of 2.5 × 10^9^ colony-forming units (CFU) (Additional file 1: Figure S1d). These results suggested that a phage-displayed Nb library targeting CD47 was successfully constructed.

To screen CD47-specific Nbs, four consecutive rounds of phage display bio-panning were conducted, after which the enrichment fold of CD47-specific VHHs has been reached to 19 times (Fig. 1b). 9 anti-CD47 Nbs with different sequences of amino acid in complementary determining region 3 (CDR3) were selected through PE-ELISA assay and sequencing analysis. These 9 Nb-IgG4 fusion proteins were expressed and purified through CHO expression system, and activities of these fusion proteins blocking CD47 were then confirmed by FACS. As indicated in Fig. 1c, Nb1-IgG4 interrupted 95% interaction between hCD47 and SIRPα. Hence, Nb1-IgG4 was selected as the candidate CD47-specific functional Nb fusion protein for further study.

### Characterization of anti-CD47 Nb fusion protein HuNb1-IgG4

**Fig. 2:**
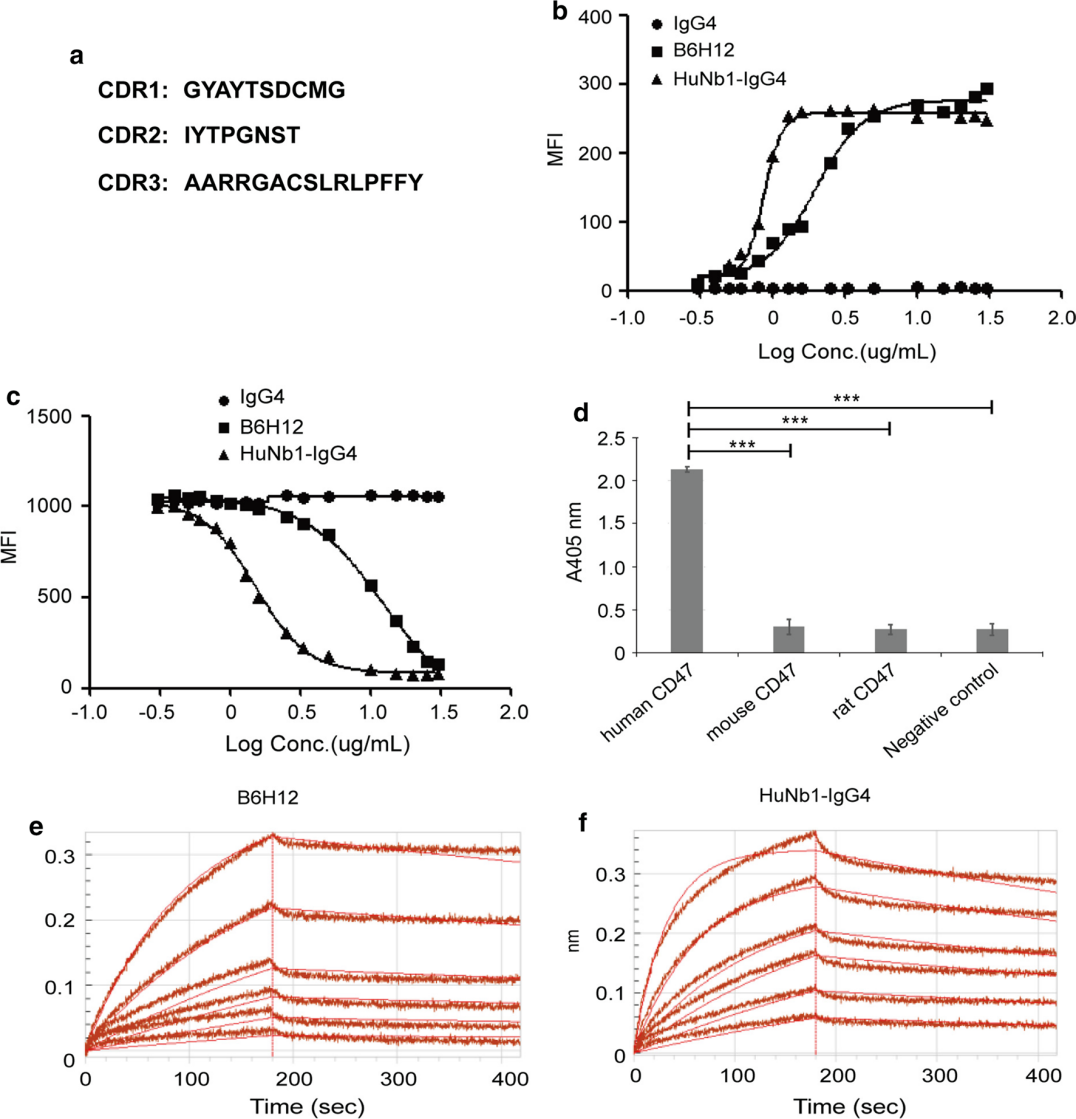
Characterization of humanized Nb1-IgG4 (HuNb1-IgG4). **a** CDRs sequences of HuNb1-IgG4 were shown. **b** The activity of HuNb1-IgG4 binding to hCD47 on the surface of cells was determined by FACS. **c** The activity of HuNb1-IgG4 blocking CD47/SIRPα was determined by FACS. **d** The specificity of HuNb1 to human CD47, mouse CD47 and rat CD47 antigen was detected by ELISA. The above experiments were performed in triplicate and data were presented as mean ± SD. ***P < 0.001 versus control. **e** The affinity of B6H12 and HuNb1-IgG4 to CD47 was detected by Fortebio detection. The experiment was performed in triplicate and one representative experiment was shown

To obtain a Nb with clinical therapeutic potential, Nb1-IgG4 was humanized (designated as HuNb1-IgG4) to minimize immunogenicity. The CDR sequences of HuNb1-IgG4 were displayed in Fig. 2a. To determine characterization of HuNb1-IgG4, the binding and blocking activity targeting CD47 were first determined by FACS. The results showed that HuNb1-IgG4 exhibited higher CD47 binding activity than B6H12, a known CD47 function-blocking monoclonal antibody (EC_50_: 0.868 μg/mL vs 1.948 μg/mL) (Fig. 2b, Table 1). In line with this, HuNb1-IgG4 had higher inhibitory effect against CD47 compared to B6H12 (IC_50_: 1.509 μg/mL vs 12.341 μg/mL) (Fig. 2c, Table 1).

**Table 1:** Comparison of blocking effect and CD47 reporter stimulation effect between B6H12 and HuNb1-IgG4

	B6H12	HuNb1-IgG4
Binding effect (EC_50_)	1.948 μg/mL	0.868 μg/mL
Blocking effect (IC_50_)	12.341 μg/mL	1.509 μg/mL
Affinity (KD)	7.81E−09 M	4.85E−09 M

*EC*_*50*_ 50% effective concentration, *IC*_*50*_ 50% inhibitory concentration, *KD* equilibrium dissociation constant

We next determined other characterization of HuNb1-IgG4 including specificity and affinity. The ELISA detection result demonstrated that HuNb1 specifically binds to human CD47 but not to other species of CD47, including rat CD47 and mouse CD47 (Fig. 2d). In the affinity assay, we showed that the KD value of HuNb1-IgG4 was lower than that of B6H12 (4.85E−09 M vs 7.81E−09 M), which implies HuNb1-IgG4 exhibits higher affinity compared with B6H12 (Fig. 2e, f, Table 1). These results suggest that HuNb1-IgG4 exhibits high specificity and affinity.

### HuNb1-IgG4 inhibits tumor growth through inducing tumor cell phagocytosis

**Fig. 3:**
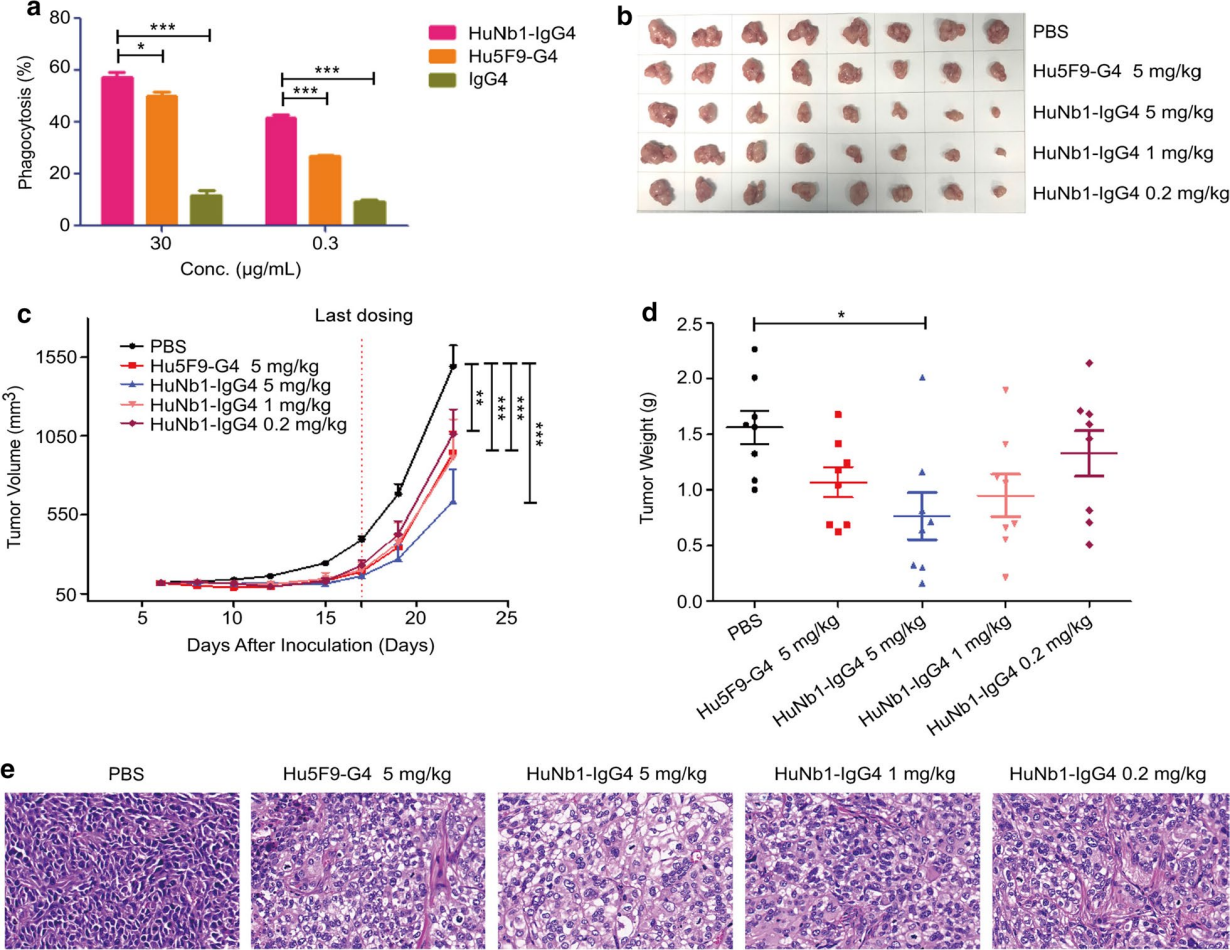
HuNb1-IgG4 induces phagocytosis and shows high activity of anti-tumor in human ovarian tumor-engrafted mice models. **a** Representative result for phagocytosis of CFSE-labeled Jurkat E6.1 cells phagocytosed by macrophages. The results were analyzed by FACS and showed in a bar graph. The experiment was performed in triplicate and data were presented as mean ± SD. **b** BALB/c nude mice were subcutaneously transplanted with SKOV3 cells and treated with low (0.2 mg/kg), medium (1 mg/kg) and high (5 mg/kg) dose of HuNb1-IgG4 and high (5 mg/kg) dose of Hu5F9-G4 or PBS as the control (n = 8). Tumor tissues from all mice in each group were shown. **c** Tumor volumes were measured and the average volume is shown. **d** Tumor weights were measured and shown in the graph. **e** H&E staining of SKOV3 tumors from control mice and mice treated with 5 mg/kg Hu5F9-G4 or different dose of HuNb1-IgG4. Magnification of ×400. The above animal experiments were performed in triplicate and one representative experiment was displayed. Data were presented as mean ± SEM. *P < 0.05, **P < 0.01, ***P < 0.001 versus control

The mechanism of CD47-targeted therapies mainly involves phagocytosis induced by macrophages. Hence, we investigated whether HuNb1-IgG4-mediated blockade of CD47 enhanced macrophage-mediated phagocytosis. The results of FACS assay indicated that HuNb1-IgG4 induced phagocytosis of Jurkat E6.1 cells (Fig. 3a). Moreover, the extent of phagocytosis in the group treated with HuNb1-IgG4 was greater than that in the group treated with Hu5F9-G4, a typical representative targeting CD47 developed in clinical stage.

Next, we evaluated the anti-tumor effects of HuNb1-IgG4 in SKOV3 cells xenograft models. The results showed that SKOV3 tumor growth was significantly inhibited in a dose-dependent manner in mice treated with HuNb1-IgG4 (Fig. 3b), compared with PBS-treated control mice. Of note, the inhibitory effect on tumor growth in the group with 5 mg/kg HuNb1-IgG4 treatment was even more obvious than that in the group with 5 mg/kg Hu5F9-G4 treatment, in terms of tumor volume and tumor weight (Fig. 3c, d). Tumor growth inhibition was in line with the results of tumor tissue H&E staining. Tumor cells in PBS-treated group showed intact structure and regular shape while tumor cells in HuNb1-IgG4-treated group exhibited apoptosis and necrosis in a dose-dependent manner. The damage extent of tumor cells in 5 mg/kg HuNb1-IgG4 treatment was even greater than that in the group with 5 mg/kg Hu5F9-G4 (Fig. 3e). Similarly, in the lymphoma mouse model, we observed that HuNb1-IgG4 treatment resulted in apoptosis and necrosis of tumor cells and enhanced macrophage infiltration into tumor tissue compared to PBS treatment (Additional file 2: Figure S2), suggesting that HuNb1-IgG4 induced tumor cell phagocytosis mediated by macrophage.

### HuNb1-IgG4 shows high safety for RBCs

**Fig. 4:**
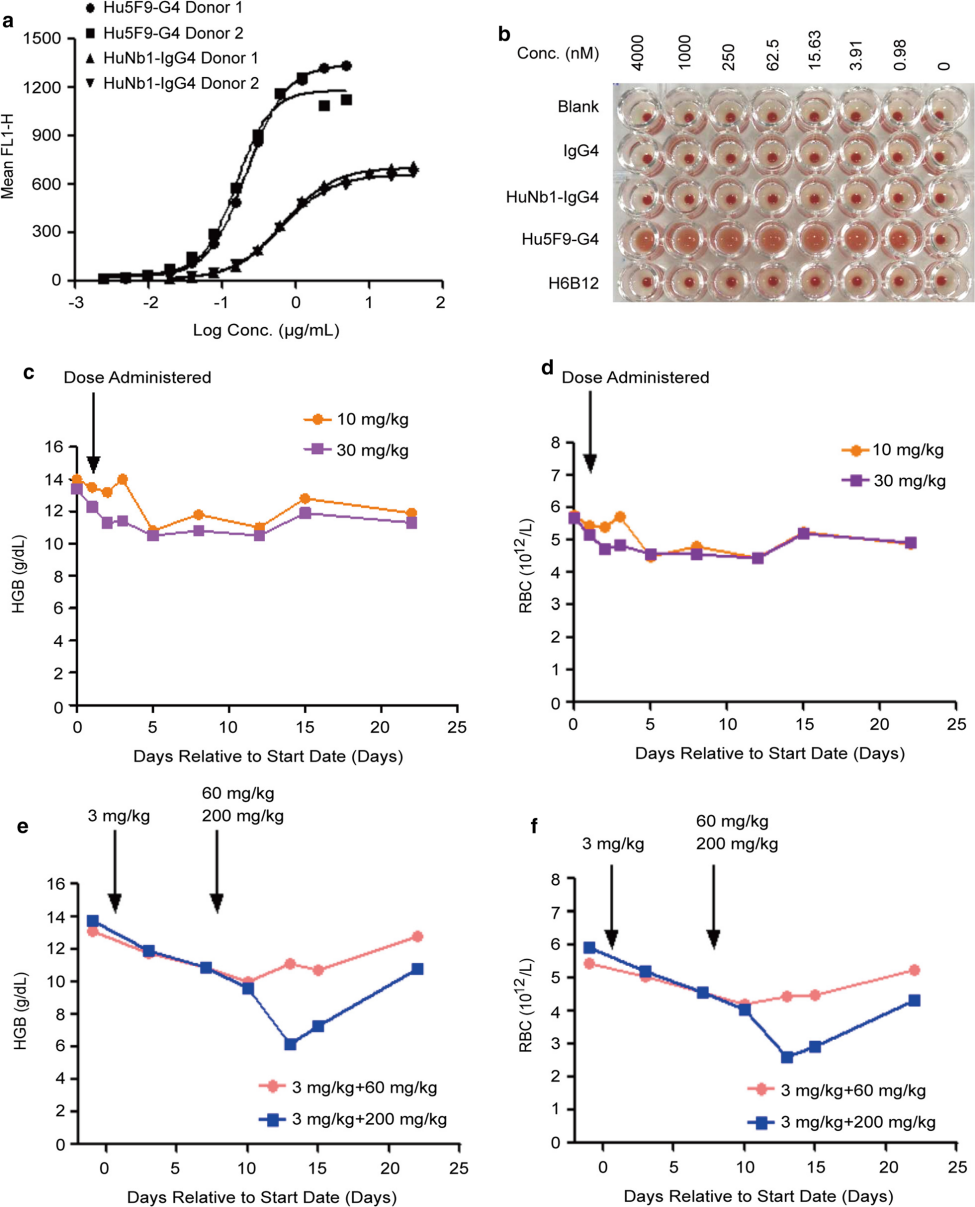
HuNb1-IgG4 shows low affinity to human RBCs and high safety in cynomolgus monkeys. **a** The activity of HuNb1-IgG4 and Hu5F9-G4 binding to RBCs isolated form fresh blood of six people. The binding activity was detected by FACS. The binding activity detections from two people were shown. The result from other four people were displayed in Additional file 3. **b** The effect of HuNb1-IgG4 on RBC agglutination, with Hu5F9-G4 and H6B12 as controls. The experiment was performed in triplicate and one representative experiment was shown. **c**, **d** The HGB levels (**c**) and RBC counts (**d**) in two individual cynomolgus monkeys administrated with a single intravenous infusion of HuNb1-IgG4 at the doses of 10 mg/kg or 30 mg/kg. **e**, **f** The HGB levels (**e**) and RBC counts (**f**) in two individual cynomolgus monkeys administrated with a priming dose of 3 mg/kg followed by another dose of 60 mg/kg or 200 mg/kg respectively. The above monkey experiments were performed once

To assess whether HuNb1-IgG4 treatment elicited adverse effects on RBCs, binding activity assay and RBCs agglutination assay were performed. As indicated in Fig. 4a and Additional file 3: Figure S3, the binding activity of HuNb1-IgG4 to RBCs from six donors was much lower than that of Hu5F9-G4. In addition, HuNb1-IgG4 had no effect on the aggregation of RBCs at concentrations ranging from 0.98 to 4000 nM, which is similar to the effect of IgG4, while Hu5F9-G4 induced serious RBC agglutination and B6H12 also induced RBC agglutination to some extent (Fig. 4b).

We next assessed the effect of HuNb1-IgG4 on hematopoietic system of cynomolgus monkeys. As shown in Fig. 4c, d, the HGB level and RBC counts were not significantly influenced with the administration of 10 mg/kg and 30 mg/kg HuNb1-IgG4 respectively. Furthermore, we found that treatment with a priming dose of 3 mg/kg followed by another dose of 60 mg/kg was well tolerated in cynomolgus monkeys. Although HGB and RBC at first were rapidly decreased in the group of 200 mg/kg treatment, they restored to the normal level after about ten days (Fig. 4e, f). In line with these findings, the body temperature and white blood cell (WBC) counts were basically remained to be normal. The WBC and Neutrophil (Neut) counts were temporary elevated after treatment with HuNb1-IgG4, while they restored rapidly to normal level (Additional file 4: Tables S1 and S2). Collectively, HuNb1-IgG4 showed no obvious adverse events in vivo, which overcomes the drawbacks of current available CD47 mAbs.

### Large scale production of HuNb1-IgG4 in CHO-S cells

**Fig. 5:**
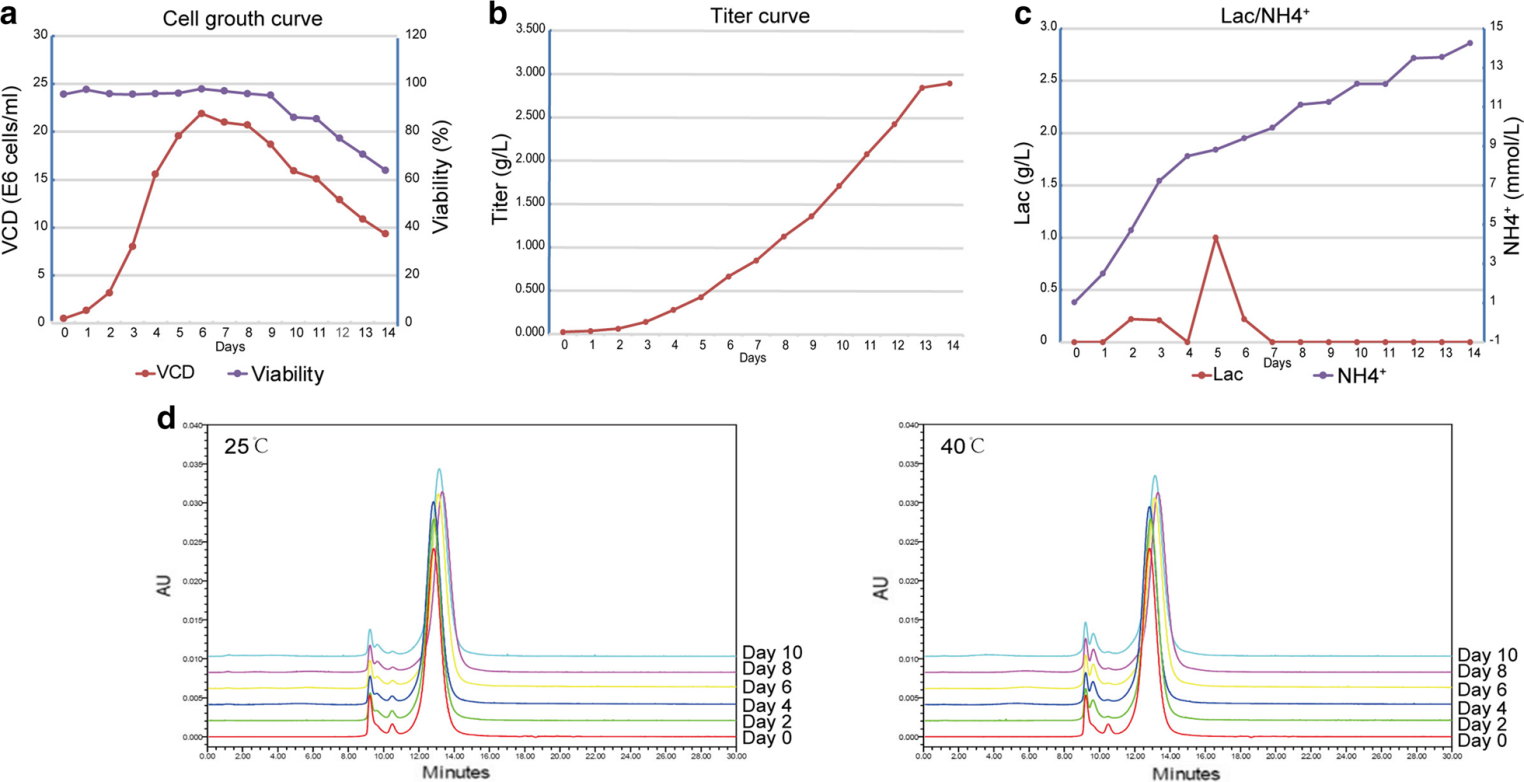
Large scale production of HuNb1-IgG4 in CHO-S cells expression system. **a** The viable cell density (VCD) and the viability were detected.** b** The protein titer was determined within 14 days. **c** The lactic acid (Lac) and NH4^+^ produced during the culture process.** d** The thermal stability of HuNb1-IgG4 was detected by SEC-HPLC detection. The experiments were performed in triplicate and one representative experiment was shown

In order to obtain HuNb1-IgG4 with high yield, HuNb1-IgG4 expression was induced in CHO-S cells growing in the fermentation tank with 40% dissolved oxygen (DO). As shown in Fig. 5a, the viable cell density (VCD) reached the highest value of about 2.2E7 cells/mL on the sixth day. Cell viability maintained 60–90% between day 5–9 while it was decreased rapidly in the late culture period (Fig. 5a). HuNb1-IgG4 protein level was increased steadily in the culture supernatant within 2 weeks, and the protein expression amount reached 2.9 g/L at Day 14 (Fig. 5b). For cell metabolism, the production of lactic acid during cell culture was extremely low, and sodium bicarbonate was not required throughout the culture process. However, the NH4^+^ concentration was gradually increased and reached 14.26 mmol/L in Day 14 (Fig. 5c). The culture condition was thus need to be slightly optimized to reduce the cumulative concentration of NH4^+^. Thermostability analysis showed that HuNb1-IgG4 was kept stable after incubation at 25 °C and 40 °C for 10 days (Fig. 5d). As shown in Table 2, the proportion of monomer form of HuNb1-IgG4 remained to be 90% approximately at 25 °C or 40 °C within 10 days. In summary, our results imply that we could obtain HuNb1-IgG4 with high activity, high yield and good thermal stability in CHO-S expression system.

**Table 2:** The proportion of monomer form of HuNb1-IgG4 under 25 ℃ and 40 ℃

	25 °C			40 °C		
	Monomer (%)	Aggregates (%)	Fragments (%)	Monomer (%)	Aggregates (%)	Fragments (%)
Day 0	88.98	10.50	0.52	88.98	10.50	0.52
Day 2	89.26	10.03	0.72	91.27	8.18	0.56
Day 4	90.18	9.74	0.08	88.91	10.65	0.44
Day 6	90.79	9.17	0.04	89.03	10.93	0.03
Day 8	91.24	8.71	0.06	88.58	10.34	1.08
Day 10	90.74	9.23	0.03	89.04	10.70	0.26

### A bispecific antibody, co-targeting CD47 and CD20, shows high anti-tumor activity

**Fig. 6:**
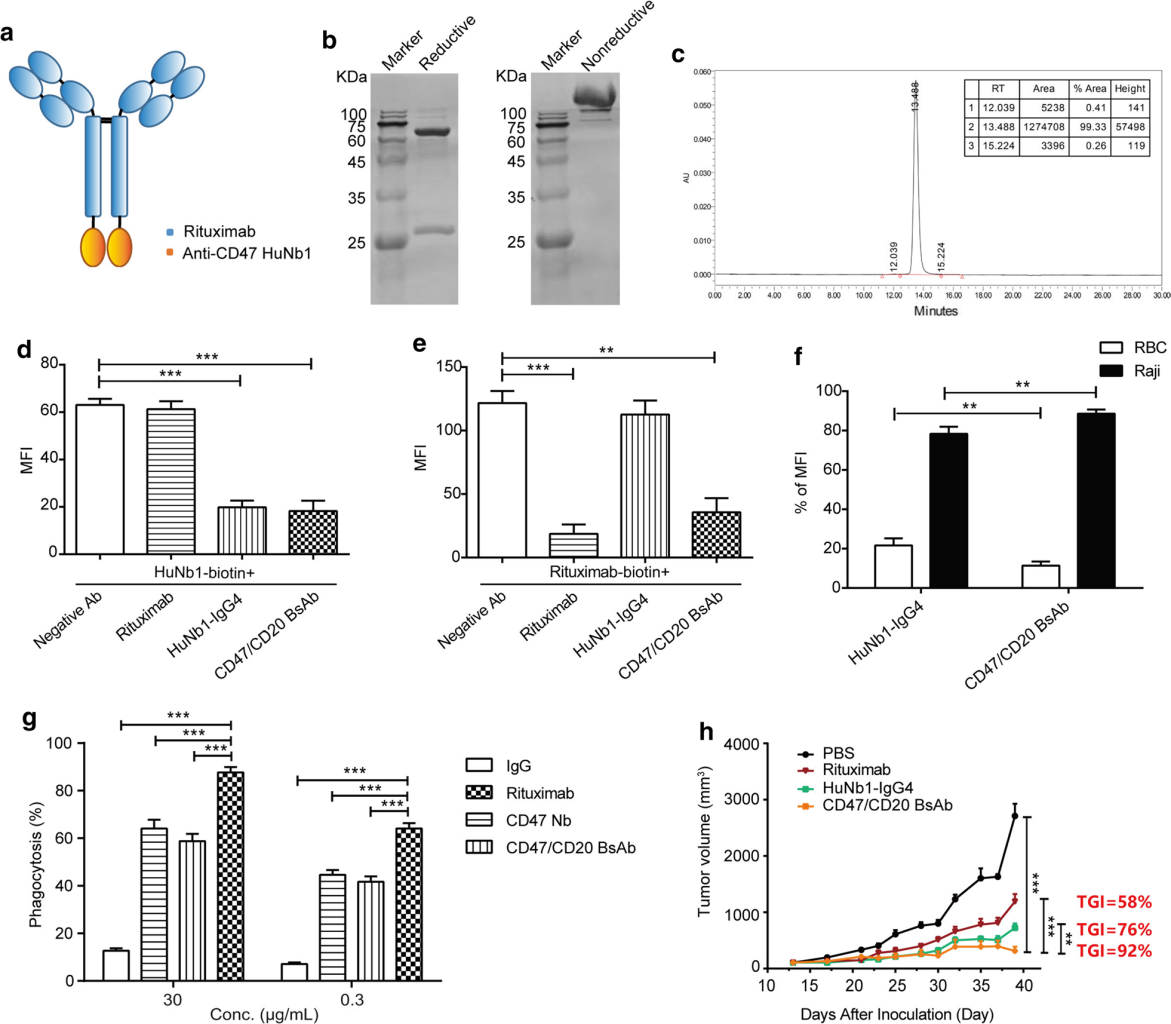
The anticancer effect of anti-CD47/CD20 bispecific antibody against lymphoma in vivo. **a** The structure of anti-CD47/CD20 bispecific antibody. **b** The reductive (left) and nonreductive form (right) of BsAb were displayed through SDS-PAGE. **c** The purity of BsAb was determined through HPLC analysis. The above experiments were performed in triplicate and one representative experiment was shown. **d**, **e** The specificity of CD47/CD20 BsAb towards CD47 **(d)** or CD20 **(e)** expressed on Raji cells. Raji cells was co-incubated with HuNb1-biotin or Rituximab-biotin as well as Negative Ab, Rituximab (CD20 mAb), HuNb1-IgG4 (CD47 Nb) or CD47/CD20 BsAb. The binding activity was detected by FACS. **f** The MFI percentage of Raji cells and RBCs. Raji cells and tenfold excess RBCs were incubated with HuNb1-IgG4 or CD47/CD20 BsAb respectively. FITC positive antibodies binding to cells were detected by FACS. The RBCs were derived from three donors. **g** CD47/CD20 BsAb induces phagocytosis of target cells. Phagocytosis of CFSE-labeled Raji cells mediated by macrophages was analyzed by FACS. The above experiments were performed in triplicate and data were presented as mean ± SD. **P < 0.01, ***P < 0.001 versus control. **h** The male NOG mice were injected through tail vein with Raji cells and treated with HuNb1-IgG4, Rituximab or CD47/CD20 BsAb at the same dose (20 mg/kg), with PBS as the control (n = 6). Tumor volumes and the index of tumor volume inhibition (TGI) were shown. The experiment was performed in triplicate and one representative experiment was shown. Data were presented as mean ± SEM. **P < 0.01, ***P < 0.001 versus control

In order to facilitate the selective binding of anti-CD47 HuNb1 to tumor cells and induce a more powerful antitumor immune response, we established anti-CD47/CD20 bispecific constructs. The structure of BsAb, which consists of Rituximab and HuNb1, is illustrated in Fig. 6a. After affinity chromatography and molecular sieve chromatography, the reductive and nonreductive form of BsAb was displayed in Fig. 6b, and the purity was 99.33% through HPLC analysis (Fig. 6c).

To determine whether CD47/CD20 BsAb bind specifically to CD47 and CD20 expressed on the surface of Raji cells, we performed the competition binding assay. The result showed that CD47/CD20 BsAb competitively bound to CD47 expressed on Raji cells, which was reflected by the decreased binding of HuNb1-biotin to CD47 (Fig. 6d). Similarly, CD47/CD20 BsAb competitively bound to CD20 expressed on Raji cells (Fig. 6e).

Normal cells, especially RBCs with high expression of CD47 might create “antigen sink” that influences the number of anti-CD47 antibodies binding to CD47 expressed on tumor cells and reduces the efficacy of their anti-tumor activities. Design of BsAbs co-targeting CD47 and CD20 might be a potential strategy to avoid the antigen sink as BsAbs exhibit stronger binding to dual antigen-expressing cells compared to single-antigen cells as a result of higher avidity interactions by multivalency binding. To confirm this, CD20^+^ CD47^+^ Raji cells were mixed with a tenfold excess of RBCs prior to incubation with BsAb or HuNb1-IgG4 and the fluorescent intensity of each cell type bound by BsAb or HuNb1-Ig4 was evaluated by FACS. We found that the mean fluorescence intensity (MFI) of Raji cells was about fourfold higher than that of RBCs in the group treated with HuNb1-IgG4 while it was about eightfold higher than that of RBCs in the group treated with CD47/CD20 BsAb. These findings suggest that both of HuNb1-IgG4 and CD47/CD20 BsAb bind preferentially to tumor cells, but CD47/CD20 BsAb showed higher preference compared to HuNb1-IgG4 (Fig. 6f).

We next assessed the biological function of CD47/CD20 BsAb including phagocytosis and anti-tumor activity. Our results demonstrated that CD47/CD20 BsAb enhanced phagocytosis of Raji cells in a dose-dependent manner. Importantly, the extent of phagocytosis induced by CD47/CD20 BsAb was greater than HuNb1-IgG4 and Rituximab (Fig. 6g). In the model of Raji cell lymphoma NOG mice, tumor growth was significantly inhibited in the groups of all three drugs treatment compared with PBS treatment. However, CD47/CD20 BsAb treatment showed the maximal inhibition of tumor growth (TGI of 92%), compared with HuNb1-IgG4 or Rituximab treated group (TGI of 76% or 58%), which is in line with the highest phagocytosis showed in the group of CD47/CD20 BsAb treatment (Fig. 6h). Taken together, these results suggested that HuNb1-IgG4 or CD47/CD20 BsAb was a promising candidate in the treatment of lymphoma.

## Discussion

The blockade of CD47 by mAbs is a promising and novel strategy for malignant hematological tumors and solid tumors [24, 25]. However, therapeutic antibodies targeting CD47 in the clinical stage would result in the on-target risk of anemia and hemagglutination [26]. To reduce this on-target risk, our goal is to develop a novel anti-CD47 nanobody with low affinity for RBCs, which does not cause platelet aggregation in human.

Although the use of mAbs has achieved remarkable success in a wide range of malignant tumors, the large size (150 kDa) of conventional mAbs is a drawback that limits their penetration into tumors [27–29]. Nbs (12 kDa) or Nb-based human heavy chain antibodies, derived from a single variable domain of camelid heavy chain antibodies, can overcome this limitation [30]. Some studies have shown deep penetration of Nbs into tumors. Especially, fusion of Nbs to human Fc-domains not only yields highly soluble and versatile heavy chain antibodies, but also combines advantages of Nbs and human Fc-domains. In the present study, we obtained 9 anti-hCD47 Nbs from the phage display library derived from immunized camel with the ECD of human CD47. Moreover, each of these 9 Nbs was designed a fusion protein consisted of Nbs and human Fc-domain of IgG4. Of the 9 Nbs-IgG4, HuNb1-IgG4 shows the highest activity of blocking the interaction between hCD47 and SIRPα. HuNb1-IgG4 is a Nb-based human heavy chain antibody combined with advantages of Nbs and human Fc domains. Not surprisingly, HuNb1-IgG4 shows better efficacy of anti-ovarian tumor and anti-lymphoma compared to anti-CD47 Hu5F9-G4 monoclonal antibody at the same doses. Additionally, Nbs are much suitable as modules to build bispecific antibodies as Nbs could be easily linked into dimers and multimers due to their completely hydrophilic surface. In this study, we constructed anti-CD47/CD20 bispecific antibody, showing the best efficacy of anti-lymphoma compared to the group treated with only Rituximab or only HuNb1-IgG4.

B6H12, an anti-human CD47 mAb, has been widely used to evaluate phagocytosis and anti-tumor activities induced by blocking CD47-SIRPα interaction. In this study, B6H12 was selected as a positive control to demonstrate HuNb1-IgG4 activities in binding to CD47 and blocking CD47-SIRPα interaction. The activities of HuNb1-IgG4 were higher than that of B6H12, especially blocking CD47-SIRPα activity of HuNb1-IgG4 (IC_50_: 1.5 μg/mL) with ninefolds higher than that of B6H12 (IC_50_: 12.3 μg/mL). Moreover, HuNb1-IgG4 exhibited much higher affinity of human CD47 than that of mouse and rat CD47. These findings suggest that HuNb1-IgG4 is a potent Nb fusion protein with high activity and specificity. Hu5F9-G4, a humanized IgG4 antibody, is the typical representative targeting CD47 developed in clinical stage [15, 31, 32]. In preclinical in vivo models, Hu5F9-G4 showed high activity against a variety of hematologic malignancies and solid [16]. Hu5F9-G4 was thus selected as a positive control and the comparative drug to show functional activities of HuNb1-IgG4 in the present study. HuNb1-IgG4 bound to CD47 with a KD of 4.85 × 10^–9^ M that is lower than KD (8 × 10^–9^ M) of Hu5F9-G4 binding to CD47 [16]. In line with this, HuNb1-IgG4-induced phagocytosis and in vivo anti-tumor effects were stronger than that of Hu5F9-G4 in the same concentration and doses.

Although Hu5F9-G4 showed good tolerance in clinical phase I, there was still a large proportion of patients with anemia (57%) and hemagglutination (36%) [26]. This is due to CD47 high expression on RBCs, especially aged RBCs and blockade of CD47 on RBCs can lead to their phagocytic removal by macrophages, which eventually induced anemia and hemagglutination [33–35]. Thus, development of antibodies targeting CD47 with low affinity to RBCs is an important direction. In the present study, affinity of HuNb1-IgG4 binding to RBCs was over tenfolds lower than that of Hu5F9-G4 to RBCs. Consistent with this, in the whole blood assay, HuNb1-IgG4 did not resulted in the aggregation of RBCs while Hu5F9-G4 caused RBCs aggregation at the same range of doses. In non-human primate study, a dose-dependent drop in RBC number and HGB were observed in response to HuNb1-IgG4. However, the minimum level of HGB, which appeared on study day 5, was still above 10 g/dL in response to 10 or 30 mg/kg HuNb1-IgG4. In the previous study, 30 mg/kg single dose Hu5F9-G4 could lead to less than 10 g/dL of HGB in the monkey [16], which represents risk of anemia or asks for blood transfusion to restore RBC function. Therefore, HuNb1-IgG4 shows better safety in view of RBCs protection compared with Hu5F9-G4, which is due to lower affinity of HuNb1-IgG4 to RBC. Similar with our present findings, AO-176, a novel humanized CD47 mAb, has been recently shown negligible binding to human RBCs [36]. Although the mechanisms by which HuNb1-IgG4 and AO-176 have lower affinity for human erythrocytes are unclear, these findings clearly indicate that antibodies or Nbs preferentially binding to tumor versus normal cells can provide better safety.

CD47 expression is unregulated on tumor cells, but its ubiquitous expression in normal tissues may create an ‘antigen sink’ that could decrease the therapeutic effects of anti-CD47 antibody. We herein showed that HuNb1-IgG4 blocking CD47/SIRPα had over tenfold lower affinity to RBCs relative to Hu5F9-G4, suggesting that HuNb1-IgG4 should reduce “antigen sink” created by CD47 ubiquitous expression. However, HuNb1-IgG4 still showed binding activity, albeit much low, for RBCs, which might be still a risk factor contributing to adverse events in hematopoietic system. Improved selectivity of antibodies targeting CD47 expressed on tumor cells should be important for development of successful antibody drugs. BsAb represents a feasible strategy to achieve this goal as a result of its stronger affinity to dual antigen-expressing cells. Considering that Nbs are easy to be modified and unique feature, they are ideal modules to construct BsAbs. We thus constructed a BsAb consisted of HuNb1 and Rituximab, co-targeting CD20 and CD47. Importantly, this BsAb was shown preferential binding to Raji cells versus RBCs in our study. Not surprisingly, the synergized anti-tumor activities of this BsAb were observed in NOG mice. This synergy, however, was the result of CD20 blockade, not decreased antigen sink of CD47 considering that HuNb1 does not bind to CD47 expressed in mice. Thus, this synergy by our CD20/CD47 BsAb might be more obvious in human body compared to treatment of HuNb1, Rituximab or their combination. Also, safety of our CD20/CD47 BsAb in human study might be better as a result of significant decreased binding to normal cells relative to single combination of HuNb1 and Rituximab.

## Conclusions

In this work we reported a novel Nb fusion protein HuNb1-IgG4 targeting CD47, showing potent anti-tumor activities and high safety for hematopoietic system in the preclinical studies. Potent anti-tumor activities were associated with macrophage-mediated phagocytosis induced by HuNb1-IgG4, which is due to blockade of CD47/SIRPα. High safety is due to lower affinity of HuNb1-IgG4 to human RBCs, thereby avoiding RBCs aggregation, which is an important advantage compared to CD47-targeting mAbs developing in clinical stage. To further improve the preference of antibodies binding to tumor cells, we constructed anti-CD47/CD20 BsAb consisted of HuNb1 and Rituximab, showing stronger anti-tumor activity in vivo compared to HuNb1 and Rituximab. considering that pharmacokinetics and biodistributions of HuNb1-IgG4 and the BsAb in primates are of importance for dose selection in future clinical studies, they should be further investigated in the primates. Taken together, our study provides a novel anti-CD47 Nb preferentially binding to tumor cells, with potential as a single drug or an ideal module to build BsAb.

## Supplementary information

**Additional file 1: Figure S1.** Library construction.

**Additional file 2: Figure S2.** The IHC staining of HuNb1-IgG4 on macrophage in lymphoma mouse model.

**Additional file 3: Figure S3.** The activity of HuNb1-IgG4 and Hu5F9-G4 binding to RBCs isolated from fresh blood of four people.

**Additional file 4: Tables S1 and S2.** The body temperature and different type cells percentage in WBC of cynomolgus monkeys administered with HuNb1-IgG4 at the doses of 3+60 mg/kg and 3+200 mg/kg.

## Data Availability

All data generated or analyzed during this study are included in the article and additional file.

## Abbreviations

Nbs: nanobodies
RBCs: red blood cells
BsAb: bispecific antibody
SIRPα: signal regulatory protein alpha
AML: acute myeloid leukemia
NHL: non-Hodgkin lymphoma
mAbs: monoclonal antibodies
ATCC: American Type Culture Collection
FCS: fetal calf serum
ECD: ectodomain
PBLs: peripheral blood lymphocytes
VHH: the variable domains of the heavy-chain antibodies
PE-ELISA: Periplasmic Extract ELISA
SEC-HPLC: size exclusion chromatography-high performance liquid chromatography
FACS: flow cytometry
BLI: biofilm interferometry
KD: equilibrium dissociation constant
BSA: bovine serum albumin
PBMCs: peripheral blood mononuclear cells
M-CSF: macrophage colony-stimulating factor
H&E: hematoxylin and eosin
IHC: immunohistochemistry
HRP: horseradish peroxidase
DAB: diaminobenzidine
HGB: hemoglobin
CFU: colony-forming units
CDR: complementary determining region
WBC: white blood cell
Neut: neutrophil
DO: dissolved oxygen
VCD: viable cell density
MFI: mean fluorescence intensity
