# Supplementary material for: Preclinical development of a novel CD47 nanobody with less toxicity and enhanced anti-cancer therapeutic potential
Source: J Nanobiotechnology. 2020 Jan 13;18:12. doi: 10.1186/s12951-020-0571-2 (PMC6956557; doi:10.1186/s12951-020-0571-2)
Supplement: Supplementary file 1 — Additional file 1: Figure S1. Library construction. [file 12951_2020_571_MOESM1_ESM.docx]

**Additional file 1**


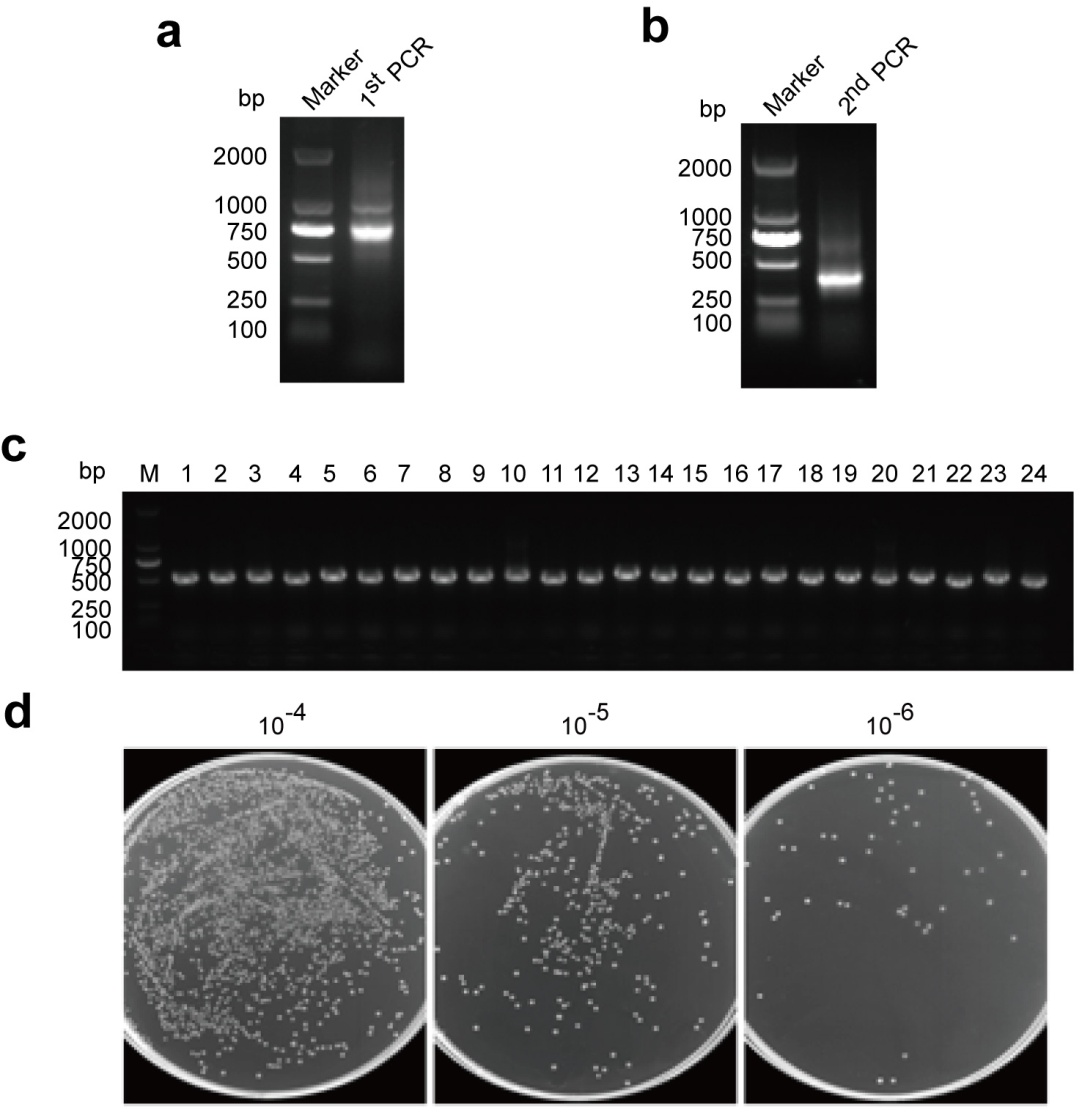


**Figure S1** Library construction. **a** The segments containing VHH gene fragments were amplified by a first PCR. **b** The fragments were amplified by a second, nested PCR. **c** Clones were randomly selected to detect the percentage of clones with a phagemid containing an insert of a proper size for a VHH. **d** Size of the library was determined by counting the number of clones after serial dilutions and plating on plates containing selective antibiotics.
