# Supplementary material for: Preclinical development of a novel CD47 nanobody with less toxicity and enhanced anti-cancer therapeutic potential
Source: J Nanobiotechnology. 2020 Jan 13;18:12. doi: 10.1186/s12951-020-0571-2 (PMC6956557; doi:10.1186/s12951-020-0571-2)
Supplement: Supplementary file 2 — Additional file 2: Figure S2. The IHC staining of HuNb1-IgG4 on macrophage in lymphoma mouse model. [file 12951_2020_571_MOESM2_ESM.docx]

**Additional file 2**


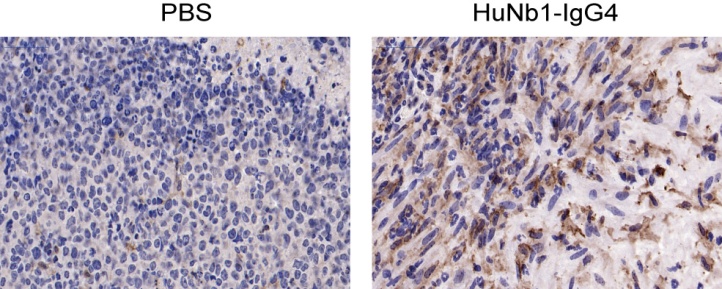


**Figure S2** The IHC staining of HuNb1-IgG4 on macrophage in lymphoma mouse model. The male NOG mice were subcutaneously transplanted with Raji cells and treated with 20 mg/kg HuNb1-IgG4 or PBS as the control (n=6). The tumor sections from mice were stained with F4/80 (brown) and one representative result was displayed.
