# Supplementary material for: Preclinical development of a novel CD47 nanobody with less toxicity and enhanced anti-cancer therapeutic potential
Source: J Nanobiotechnology. 2020 Jan 13;18:12. doi: 10.1186/s12951-020-0571-2 (PMC6956557; doi:10.1186/s12951-020-0571-2)
Supplement: Supplementary file 3 — Additional file 3: Figure S3. The activity of HuNb1-IgG4 and Hu5F9-G4 binding to RBCs isolated from fresh blood of four people. [file 12951_2020_571_MOESM3_ESM.docx]

**Additional file 3**


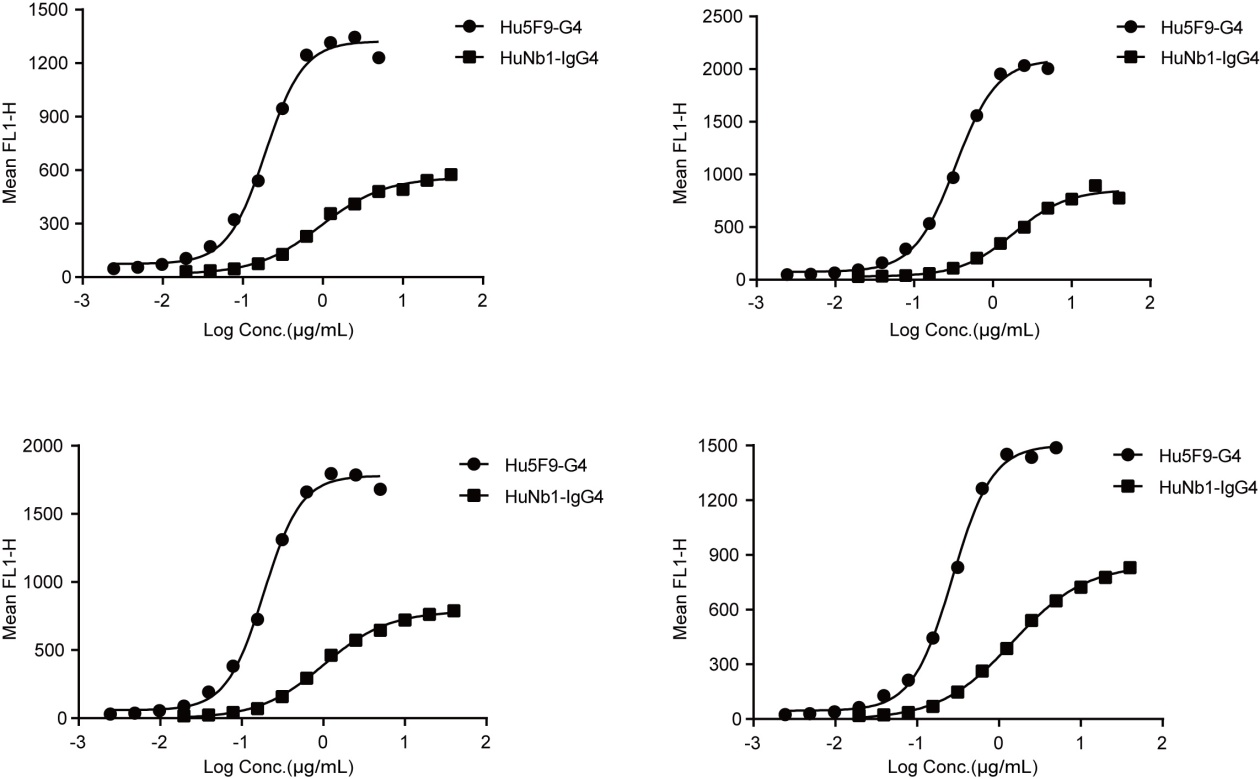


**Figure S3** The activity of HuNb1-IgG4 and Hu5F9-G4 binding to RBCs isolated from fresh blood of four people. The binding activity was detected by FACS.
