# Supplementary material for: Preclinical development of a novel CD47 nanobody with less toxicity and enhanced anti-cancer therapeutic potential
Source: J Nanobiotechnology. 2020 Jan 13;18:12. doi: 10.1186/s12951-020-0571-2 (PMC6956557; doi:10.1186/s12951-020-0571-2)
Supplement: Supplementary file 4 — Additional file 4: Tables S1 and S2. The body temperature and different type cells percentage in WBC of cynomolgus monkeys administered with HuNb1-IgG4 at the doses of 3+60 mg/kg and 3+200 mg/kg. [file 12951_2020_571_MOESM4_ESM.docx]

**Additional file 4**

**Table S1 The body temperature of cynomolgus monkeys administered with HuNb1-IgG4 at the indicated time**

|  | -1 days | 1 days | 7 days | 8 days | 15 days |
| --- | --- | --- | --- | --- | --- |
| 3+60 mg/kg | 38.8 | 38.7 | 39.1 | 39.1 | 37.8 |
| 3+200 mg/kg | 38.5 | 38.9 | 39.4 | 38.9 | 38.6 |

**Table S2 The** **percentage of different type cells in WBC of cynomolgus monkeys administered with HuNb1-IgG4 at the indicated time**

|  |  | -1 days | 3 days | 7 days | 10 days | 13 days | 15 days | 22 days |
| --- | --- | --- | --- | --- | --- | --- | --- | --- |
| WBC  (10^9/L) | 3+60 mg/kg | 12.80 | 11.60 | 17.38 | 10.23 | 11.73 | 10.89 | 10.13 |
|  | 3+200 mg/kg | 11.89 | 11.30 | 11.71 | 8.60 | 24.10 | 31.30 | 11.51 |
| Neut  (%) | 3+60 mg/kg | 32.9 | 32.8 | 46.9 | 28.9 | 28.1 | 31.5 | 22.4 |
|  | 3+200 mg/kg | 24.3 | 33.4 | 38.6 | 41.1 | 28.8 | 32.5 | 25.3 |
| Lymph  (%) | 3+60 mg/kg | 59.6 | 59.1 | 46.4 | 63.5 | 62.9 | 60.9 | 69.8 |
|  | 3+200 mg/kg | 65.9 | 57.9 | 49.9 | 49.4 | 59.6 | 59.1 | 65.9 |
| Mono  (%) | 3+60 mg/kg | 4.3 | 5.3 | 4.0 | 4.6 | 5.5 | 4.5 | 4.1 |
|  | 3+200 mg/kg | 7.2 | 6.2 | 8.7 | 5.9 | 3.7 | 4.2 | 5.0 |
| Eos  (%) | 3+60 mg/kg | 1.5 | 1.3 | 1.0 | 1.6 | 1.6 | 1.4 | 1.5 |
|  | 3+200 mg/kg | 0.7 | 0.8 | 0.8 | 0.7 | 0.8 | 0.9 | 1.3 |
| Baso  (%) | 3+60 mg/kg | 0.8 | 0.6 | 1.0 | 0.6 | 0.8 | 0.6 | 0.9 |
|  | 3+200 mg/kg | 0.8 | 0.7 | 0.6 | 0.7 | 1.8 | 2.6 | 0.9 |

Note: WBC: white blood cell; Neut: neutrophil; Lymph: lymphocyte; Mono: monocyte; Eos: eosinophils; Baso: basophil.
